# A Case of Hypercalcemia from PTHrP-Producing Fibromyxoid Sarcoma Responsive to Glucocorticoid Therapy

**DOI:** 10.1007/s00223-023-01099-8

**Published:** 2023-06-26

**Authors:** Isabella Niu, Edward C. Hsiao, Rosanna Wustrack, John J. Wysolmerski, Pamela Dann, Umesh Masharani

**Affiliations:** 1 Division of Endocrinology, Diabetes, and Metabolism, Department of Medicine, University of California, San Francisco, 400 Parnassus Ave., Suite A-550, San Francisco, CA 94143 USA; 2 Division of Endocrinology and Metabolism, Department of Medicine, Yale School of Medicine, Yale University, TAC S141D, 300 Cedar Street, New Haven, CT 06520-8020 USA

**Keywords:** Parathyroid hormone-related protein (PTHrP), Hypercalcemia, Fibromyxoid sarcoma, Glucocorticoids, Case report

## Abstract

The treatment of parathyroid hormone-related protein (PTHrP)-mediated hypercalcemia of malignancy includes treating the malignancy, intravenous fluids, and anti-resorptive therapies such as zoledronic acid or denosumab. PTHrP-mediated hypercalcemia has been reported in benign conditions such as systemic lupus erythematous (SLE) and sarcoidosis and appears to be responsive to glucocorticoids. We report a case of PTHrP-induced hypercalcemia due to a malignancy—low grade fibromyxoid sarcoma—that responded to glucocorticoid treatment. This is the first report of glucocorticoids controlling PTHrP-mediated hypercalcemia of malignancy. Immunohistochemistry of the surgical pathology localized PTHrP staining to the vascular endothelial cells within the tumor. Further studies are needed to elucidate the mechanism of glucocorticoid action in the treatment of PTHrP-mediated hypercalcemia of malignancy.

**Supplementary Information:**

The online version contains supplementary material available at 10.1007/s00223-023-01099-8.

## Introduction

Parathyroid hormone-related protein (PTHrP) is a frequent cause of hypercalcemia in solid organ malignancies such as squamous cell carcinoma, and only rarely with sarcomas [1]. Acute management of PTHrP-mediated hypercalcemia of malignancy includes intravenous fluids and antiresorptive agents [2]. PTHrP-induced hypercalcemia occasionally occurs in benign disorders such as SLE and sarcoidosis [3]. and in these cases it has been reported that the hypercalcemia is responsive to glucocorticoids [3–6].

We report an unusual case of PTHrP-mediated hypercalcemia due to malignancy (low grade fibromyxoid sarcoma [LGFMS]) which responded to glucocorticoids.

## Case Presentation

**Fig. 1:**
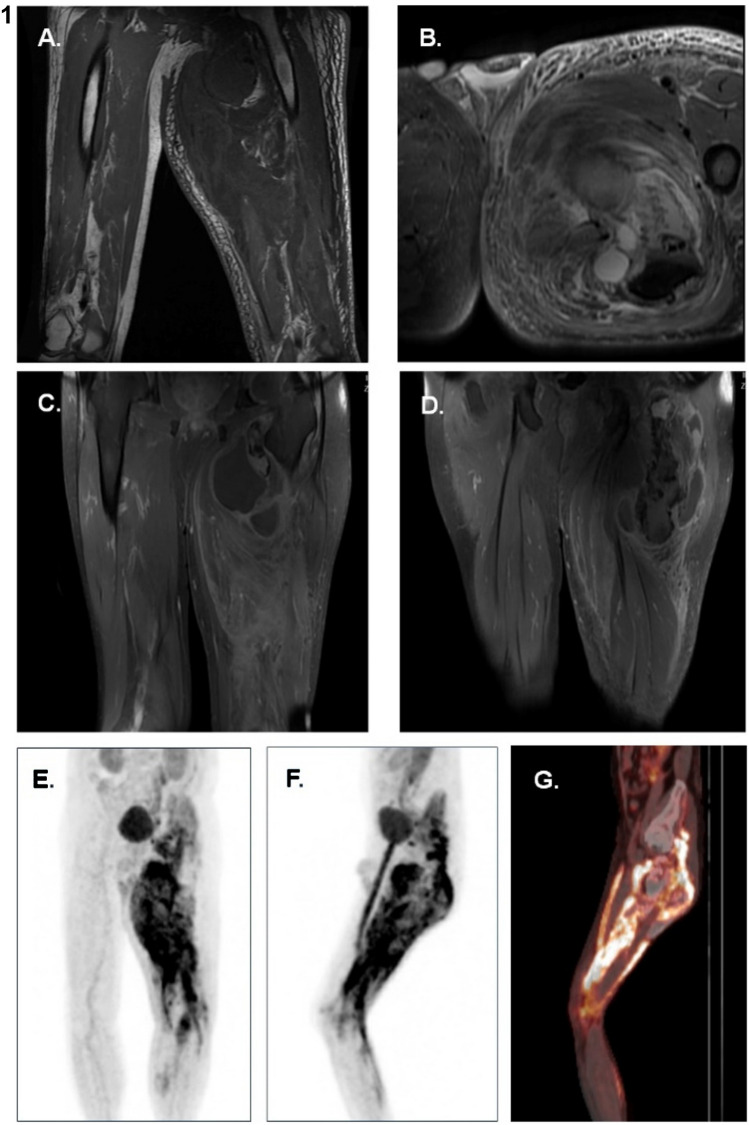
MRI Femur and FDG PET/CT Scan. MRI Femur showed a large heterogeneous mass with extensive edema and osseus matrix deposition in the left thigh, matching with areas of FDG avidity on FDG PET/CT Scan. Malignant degeneration could not be excluded, however, there was no evidence of distant metastases. **A**., **B**.: MRI Femur Without Contrast from admission, coronal and sagittal T1-weighted images. **C**., **D**.: MRI Femur With Contrast, T1-weighted images 1.5 months after initiation of methylprednisolone (3.5 months after initial presentation). **E**.,** F**., **G**.: FDG PET/CT scan from admission – **E**. and **F**. are Maximal Intensity Projection (MIP) images, and **G**. is a fused sagittal image

**Fig. 2:**
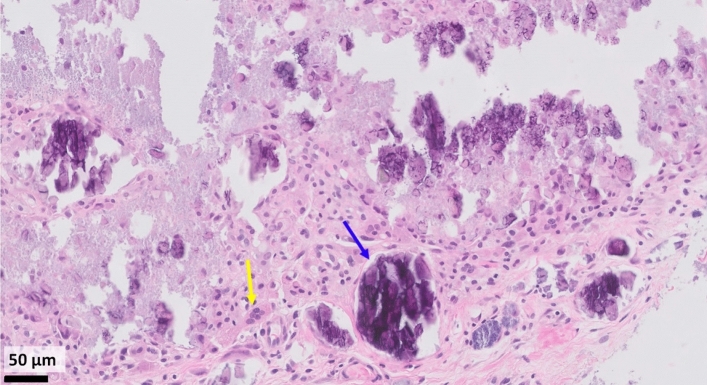
H&E stain of pathology tissue from initial biopsy. H&E slide, at 20 × magnification, notable for hydroxyapatite crystals (example shown with blue arrow) and foreign body giant cell reaction (example shown with yellow arrow). Black rectangle scale bar on bottom left corresponds to length of 50 μm

A 70-year-old man presented with pain, constipation, polyuria, confusion and swelling of a chronic left thigh mass. A ski injury 12 years prior resulted in a left thigh hematoma that was treated conservatively given its size and proximity to the sciatic nerve. The mass remained stable in size, and a diagnosis of myositis ossificans traumatica was made. Labs at admission revealed a serum calcium of 18.6 mg/dL (8.4–10.5), ionized calcium > 1.80 mmol/L (1.14–1.34), albumin 3.7 g/dL (3.4–4.8), creatinine 3.88 mg/dL (0.73–1.24; eGFR 15 ml/min), phosphorus 4.1 mg/dL (2.3–4.7), PTHrP 9.3 pmol/L (≤ 4.2), 25(OH) Vitamin D 7 ng/mL, 1,25(OH)_2_ Vitamin D 17 pg/mL (20–79), alkaline phosphatase 79 U/L (38–108), PTH 10 ng/L (18–90), TSH 1.76 mIU/L (0.45–4.12); SPEP, UPEP, and immunofixation were negative. Magnetic resonance imaging (MRI) of the leg showed a large heterogenous mass in the left adductor muscle compatible with chronic hematoma with myositis ossificans, but malignant degeneration could not be excluded. An FDG PET/CT scan showed FDG avidity and calcification in the left lower extremity with maximal hypermetabolism in the gluteus minimus and medius and no distant metastatic disease (see Fig. 1). Two biopsies of the mass showed hydroxyapatite deposition with foreign body giant cell reaction without evidence of malignancy (see Fig. 2). The second biopsy was an open biopsy to ensure that adequate material was available for complete pathological evaluation.

**Fig. 3:**
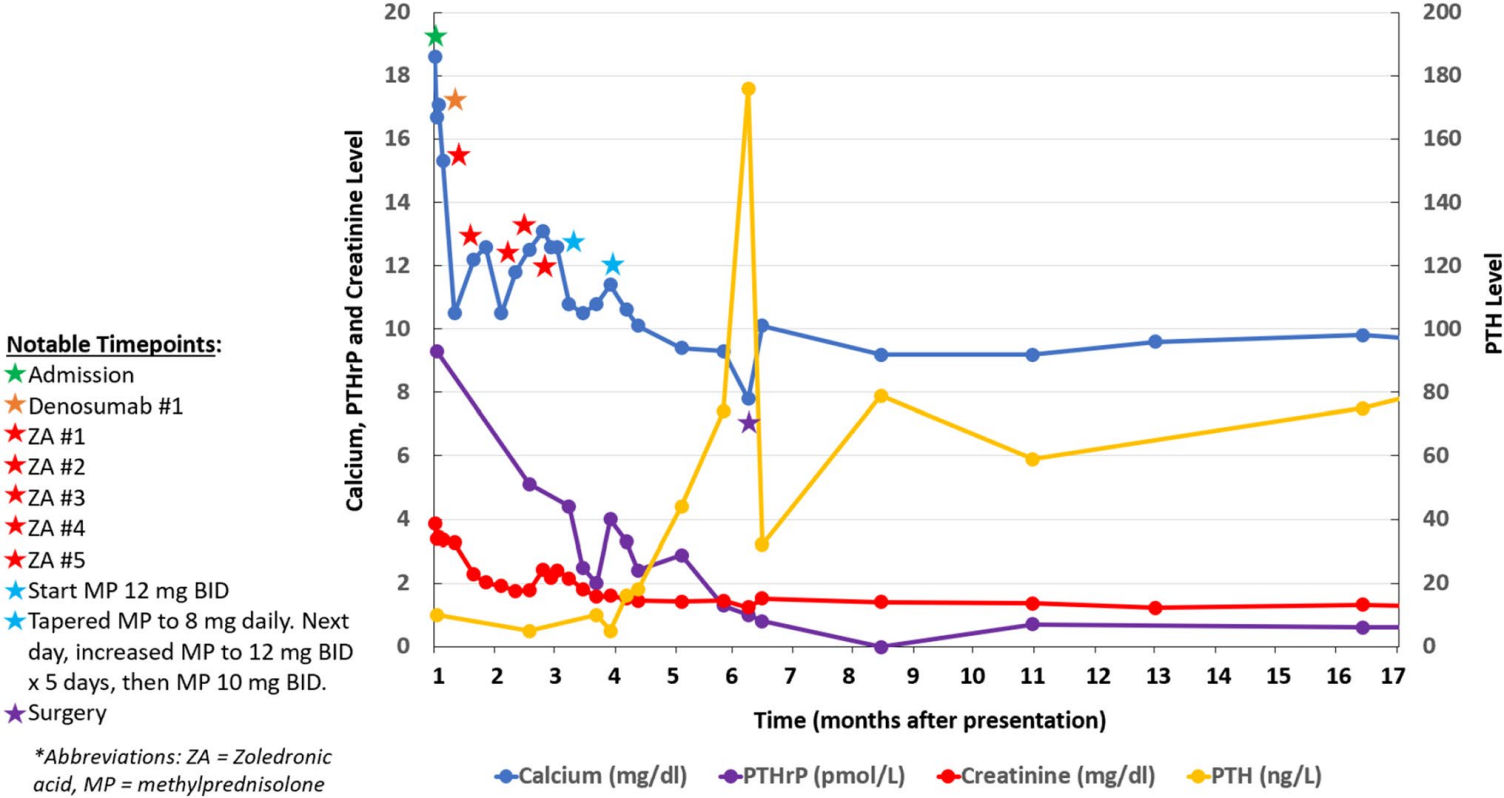
Calcium, creatinine, PTHrP and PTH trends. This figure shows the patient’s calcium, creatinine, PTHrP and PTH levels from initial admission to 10 months after surgical resection. Of note, denosumab 120 mg dose was given once on hospital day 2 after IV fluids did not lead to significant decline in calcium levels. Subsequently, IV Zoledronic acid 4 mg was given on hospital day 4, and despite multiple doses of IV zoledronic acid, calcium levels remained elevated. Once methylprednisolone was initiated, calcium levels started to normalize. When glucocorticoids were tapered, there was an increase in calcium levels, but after increasing the dose of methylprednisolone back to 12 mg twice daily (24 mg total daily) for five days, and then 10 mg twice daily (20 mg daily) afterwards, the patient maintained normal calcium and PTHrP levels until surgery. Shortly after surgery, the patient developed hypocalcemia with elevated PTH levels, but continued to have low PTHrP levels. He was treated briefly with calcium supplementation for hungry bone syndrome. Subsequent follow up calcium and PTHrP levels continue to remain normal up to the time of this publication (later results not included on graph given stability of labs and to allow improved visualization of initial biochemical trends)

**Fig. 4:**
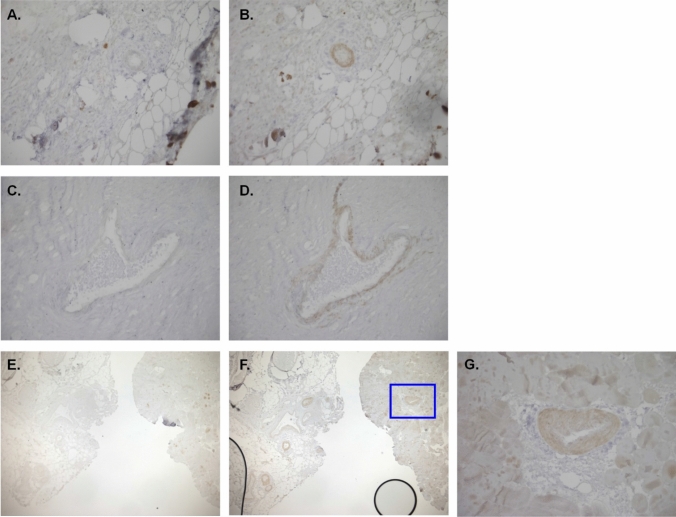
Immunohistochemistry stain of final surgical pathology tissue for IgG (negative control) and PTHrP. **A**., **B**., **C**., and **D**. are at 20 × magnification, with **A**. and **C**. showing IgG negative control stains and **B**. and **D**. showing PTHrP stains in the same window. **E**. and **F**. are at 4 × magnification, with **E**. staining for IgG negative control, and **F**. staining for PTHrP in the same window. **G**. is at 20 × magnification, a close up of the area highlighted with a blue box in **F**. and shows PTHrP staining. See Supplemental Information for more details on PTHrP Staining methods

Intravenous hydration, denosumab, and zoledronic acid initially lowered calcium to 10.5 mg/dL but the hypercalcemia proved refractory 6 weeks later (see Fig. 3). Based on the biopsy finding of hydroxyapatite deposition and foreign body giant cell reaction without evidence of malignancy, methylprednisolone 24 mg daily was started and led to a rapid decline and normalization of both the calcium and PTHrP levels. Reduction in the methylprednisolone dose resulted in a rebound increase in both PTHrP and serum calcium levels. These abnormalities reversed again on increasing the methylprednisolone dose. As chronic high-dose glucocorticoid therapy is undesirable, a decision was made to proceed with surgical resection of the mass. The hypercalcemia resolved immediately after surgery. Fifteen months after the surgery, the patient’s calcium (9.5 mg/dl) and PTHrP (0.6 pmol/L) levels remain normal. Pathology of the resected mass showed LGFMS with areas of necrosis, dystrophic calcification, and focal osseous metaplasia. Immunohistochemical studies identified PTHrP in surrounding vasculature, suggesting a possible tumor-vessel interaction (see Fig. 4).

## Materials and Methods

### Biochemical Assays

PTHrP, PTH and ionized calcium assays were performed using standard assays. More details provided in Supplemental Information.

### PTHrP Staining

Immunohistochemistry for PTHrP on FFPE sections was performed using standard techniques. Please see Supplemental Information for more details.

## Discussion

We present an unusual case of LGFMS with hypercalcemia secondary to PTHrP. To our knowledge, this is also the first report of PTHrP-induced hypercalcemia of malignancy responsive to glucocorticoid therapy.

PTHrP-mediated hypercalcemia comprises approximately 80% of cases of hypercalcemia of malignancy, most commonly in squamous cell carcinomas and rarely in sarcomas [1, 2]. The pathophysiology involves osteoclastic activation and increased calcium resorption and inhibition of phosphate reabsorption in the kidney. PTHrP, unlike PTH, does not increase renal 1α-hydroxylase activity and subsequently does not increase 1,25(OH)_2_ Vitamin D levels [2].

Since 1987, nine adult cases (and one likely case) and eight pediatric cases of PTHrP-induced hypercalcemia in patients with sarcomas have been confirmed (see Tables 1 and 2) [7–19]. In an adult case of uterine carcinosarcoma with a squamous cell carcinoma in its epithelioid component, a combination of glucocorticoids (to treat the patient’s underlying rheumatoid arthritis), intravenous fluids, diuresis, bisphosphonates and surgery lowered the calcium levels [7]. Glucocorticoids were also used in two adult cases of liposarcoma [8, 9] and one case of infantile fibrosarcoma [10]. In the first adult case, 1,25(OH)_2_ Vitamin D and PTHrP levels were elevated, and glucocorticoids were initiated to inhibit 1α-hydroxylase [8]. The patient also was given pamidronate with resolution of hypercalcemia. PTHrP level was not measured again, and the patient died soon after. In the second case, the individual was simultaneously given intravenous fluids, furosemide, and glucocorticoids with lowering of calcium. There was no measurement of PTHrP, and the individual died soon afterwards. The PTH level however was low and there were no metastases, so the authors speculated that the patient had PTHrP-mediated hypercalcemia [9]. In the pediatric fibrosarcoma case, glucocorticoids were given in combination with rehydration and diuretics to lower calcium levels. The infant died soon afterward from complications of surgery [10]. In all these cases, glucocorticoids were used in combination with other therapies, and their exact contribution in lowering calcium levels was not known. As no case included a repeat PTHrP level, it is not known if there was a direct PTHrP-lowering effect with glucocorticoid treatment.

**Table 1:** Cases of PTHrP-mediated hypercalcemia in adult patients with sarcoma

#	References	Primary tumor	Age/gender	Mechanism	Treatment/outcome
1	Nagata et al. [11]	Leiomyosarcoma	62 yo F	PTHrP (radioimmunoassay on wet tissue)	Oral fluids, calcitonin, chemotherapy. Developed pancreatitis and hypocalcemia, then died
2*	Cross and Enoch [9]	Myxoid liposarcoma	72 yo F	Likely PTHrP (suppressed PTH, bone scan without metastatic bone disease)	IV fluids, hydrocortisone, furosemide, calcitonin. Died 21 days after admission
3	Oleffe et al. [12]	Synoviosarcoma	23 yo F	PTHrP (serum)	IV fluids, IV Ibandronate, radical surgery
4	Tang et al. [13]	Leiomyosarcoma	61 yo F	PTHrP (serum, however upper end of normal range), 1,25 OH D (serum, elevated)	1st: IV fluids, diuretics, IV pamidronate, calcitonin. Hypercalcemia spontaneously resolved after 3 mo. 2nd (years later): hypercalcemia resolved 10 min after tumor resection (PTHrP became undetectable)
5	Florez et al. [14]	Rhabdomyosarcoma	71 yo M	PTHrP (normal serum level, but PTHrP-positive staining of cells on periphery of tumor)	1st: IV fluids, pamidronate 2nd: IV zoledronic acid. Patient died from disease 6 months after diagnosis
6*	Takamatsu et al. [7]	Uterine carcinosarcoma (with SCC in epithelial component)	70 yo F	PTHrP (serum)	IV fluids, diuresis, bisphosphonate, surgery. Received prednisone for rheumatoid arthritis symptoms
7	Donovan et al. [1]	Myxoid liposarcoma	Did not report	PTHrP (serum)	No details provided regarding clinical course and treatment (paper is retrospective case series)
8	Motilal Nehru et al. [15]	Endometrial stromal sarcoma	53 yo F	PTHrP (serum)	IV fluids, pamidronate, calcitonin, hemodialysis, and denosumab. Surgery, chemotherapy, and palliative radiation. Patient died from complications of malignancy
9	Jensen and Wang [16]	Angiosarcoma	72 yo M	Primary hyperparathyroidism, and PTHrP (serum)	1st: IV fluids, cinacalcet, calcitonin, parathyroidectomy 2nd: IV fluids, IV pamidronate, calcitonin. Patient transitioned to hospice
10*	Kim et al. [8]	Liposarcoma	89 yo F	1,25 OH D and PTHrP (serum)	1st: IV fluids, IV pamidronate 2nd: IV fluids, calcitonin, IV pamidronate, prednisone. Patient transitioned to hospice

Note: "1st" indicates first admission, "2nd" indicates second admission

^*^Indicates a case in which glucocorticoids were part of the patient’s treatment

**Table 2:** Cases of PTHrP-mediated hypercalcemia in pediatric patients with sarcoma

#	References	Primary tumor	Age/gender	Mechanism	Treatment/outcome
1	Lakhdir et al. [17]	Hepatic sarcoma	3 mo M	PTHrP (serum)	IV fluids, furosemide. Patient died 5 days after hospitalization
2*	Michigami et al. [10]	Infantile fibrosarcoma	6 mo M, born at 36 weeks GA	PTHrP (serum, + staining of tissue)	Rehydration, diuretics, glucocorticoids. Patient died during surgery 1 month later
3	Kawasaki et al. [18]	Rhabdomyosarcoma	3 cases in 93 total children with rhabdo-myosarcoma reviewed; ages 4 yo 5 mo to 16 yrs 7 mo, no further details	PTHrP (serum)	IV fluids, furosemide, chemotherapy, may have also been treated with calcitonin and plicamycin
4	Inoue et al. [19]	Nonrhabdomyosarcoma soft tissue sarcoma	3 mo M	PTHrP (serum)	Tumor resection, chemotherapy, but tumor continued to grow, developed complications and patient eventually died
5	Hirschfeld et al. [31]	Infantile fibrosarcoma	Full term F, diagnosed at birth	PTHrP (serum)	IV fluids, furosemide, chemotherapy, and surgical resection
6	Hirschfeld et al. [31]	Infantile fibrosarcoma	Full term M, diagnosed at birth	PTHrP (serum)	IV fluids, furosemide, chemotherapy

^*^Indicates a case in which glucocorticoids were part of the patient’s treatment

PTHrP-induced hypercalcemia has not been previously reported in individuals with LGFMS. This rare sarcoma subtype represents fewer than 5% of all soft-tissue sarcomas and often occurs in the extremities and trunk of young adults [20]. Although typically indolent and characterized by slow growth of a painless soft tissue mass and relatively benign histologic appearance, LGFMSs have a high potential to metastasize. Complete excision does not prevent local recurrence and distant metastases can occur years after the primary surgery (median 5 years, range 0 to 45 years) [20–22].

Immunohistochemistry staining of our pathology slides for PTHrP demonstrated concentration of PTHrP-positive cells in the vasculature rather than within the tumor cells. PTHrP has been proposed to have a physiologic role in vascular endothelial cells, acting as a vasodilator [23]. It has also been proposed that PTHrP may promote tumor-induced angiogenesis by increasing expression of pro-angiogenic factors such as vascular endothelial growth factor and Factor VIII, or via PTH1R activation and cAMP signaling [24]. Angiogenesis plays an important role in tumor persistence and growth, and it is possible that PTHrP expression in the endothelial cells of our patient promoted tumor growth and survival.

PTHrP-mediated hypercalcemia has been described in some benign disorders [1, 3–6]. In two cases of patients with SLE and elevated PTHrP levels, the hypercalcemia responded to glucocorticoid treatment [3, 4]. Hypercalcemia with elevated PTHrP levels has also rarely been reported in sarcoidosis [3, 5, 6]. PTHrP immunoreactivity and/or mRNA in the cytoplasm of sarcoid macrophages and multinucleated giant cells were observed in more than half of lymph node biopsies in patients with sarcoidosis TNF-α and IL-6, may stimulate PTHrP production in sarcoid macrophages [6]. In vitro studies indicate that glucocorticoids may directly decrease gene expression of PTHrP [25–27]. Hydrocortisone inhibited PTHrP gene expression when added to human carcinoma cell lines that constitutively produce PTHrP [25].

In our patient, the presence of foreign body giant cell-like reaction on the biopsy, the likelihood of an inflammatory milieu, and elevated PTHrP levels led us to initiate glucocorticoid treatment. We observed a rapid normalization of calcium, PTHrP, and PTH levels. Glucocorticoid dose reduction, however, caused an increase in PTHrP levels and hypercalcemia recurrence. This prompted surgical mass resection and the discovery that the patient had a LGFMS.

It is unlikely that the hypercalcemia in this case was mediated via 1α-hydroxylase activation. Even though giant cells were present in the initial biopsies, there were no granulomas observed in the biopsies or in the surgical pathology. Also, the 1,25(OH)_2_ Vitamin D level was low, most likely because of the suppressed PTH level. The pattern of low 1,25(OH)_2_ Vitamin D, suppressed PTH, and elevated PTHrP levels is the pattern observed in PTHrP-mediated humeral hypercalcemia [28]. In contrast, in 1,25(OH)_2_ Vitamin D-mediated hypercalcemia in granulomatous disorders, both the PTH and PTHrP levels are low [29, 30]. In this patient, the PTHrP levels were elevated and decreased in conjunction with calcium levels in response to glucocorticoid treatment and remained low after surgery. This leads us to conclude that the hypercalcemia was mediated via PTHrP action. We hypothesize that the tumor induced an increase in inflammatory cytokines and an increase in expression of PTHrP in the vascular endothelium, leading to hypercalcemia. Glucocorticoids are effective in lowering inflammatory cytokine levels and may explain why the tumor-induced hypercalcemia responded to glucocorticoid therapy.

A similar mechanism may operate in other solid tumors with PTHrP-mediated hypercalcemia and elevated inflammatory cytokine levels, and a trial of glucocorticoid therapy may be considered if standard treatments are ineffective.

## Conclusion

We report a case of PTHrP-mediated hypercalcemia responsive to glucocorticoid therapy in a patient with a LGFMS. Histochemistry showed PTHrP staining in the tumor vasculature. Additional studies are needed to determine the mechanisms underlying the beneficial effect of glucocorticoids on calcium levels. This case report raises the intriguing possibility that PTHrP-mediated hypercalcemia in other solid tumors might also be responsive to glucocorticoid therapy.

## Supplementary Information

Below is the link to the electronic supplementary material.

Supplementary file1 (DOCX 24 KB)
