# Supplementary material for: A Case of Hypercalcemia from PTHrP-Producing Fibromyxoid Sarcoma Responsive to Glucocorticoid Therapy
Source: Calcif Tissue Int. 2023 Jun 26;113(2):246–53. doi: 10.1007/s00223-023-01099-8 (PMC10372127; doi:10.1007/s00223-023-01099-8)
Supplement: Supplementary file 1 — Supplementary file1 (DOCX 24 KB) [file 223_2023_1099_MOESM1_ESM.docx]

**Supplementary Information**

**Article Title:** A Case of Hypercalcemia From PTHrP-Producing Fibromyxoid Sarcoma Responsive to Glucocorticoid Therapy

**Authors and Affiliations:** Isabella Niu, MD^1^; Edward C. Hsiao, MD, PhD^1^; Rosanna Wustrack, MD^1^; John J. Wysolmerski, MD^2^; Pamela Dann^2^; Umesh Masharani, MB, BS^1*^

^1^Division of Endocrinology, Diabetes, and Metabolism, Department of Medicine, University of California, San Francisco, CA, USA.

^2^Division of Endocrinology and Metabolism, Department of Medicine, Yale University, New Haven, CT, USA.

**Journal Name:** Calcified Tissue International

**Corresponding Author email address:**

Isabella Niu (isabella.niu@gmail.com)

Contents:

1. Biochemical assays
2. PTHrP staining
3. Additional references accompanying Table 1
4. Biochemical Assays:

All laboratory testing was collected and performed at the University of California, San Francisco (UCSF) laboratory facilities other than PTHrP, which was collected at UCSF, and processed at Mayo Medical Laboratories. Details regarding methodology for select tests listed below. Additional details regarding other blood tests are available upon request.

- Parathyroid hormone: Chemiluminescent microparticle immunoassay (Abbott Architect i2000), reference range 18-90 ng/L.
- Parathormone related protein: Immunochemiluminometric assay, reference range </= 4.2 pmol/L, processed at Mayo Medical Laboratories.
- Ionized calcium: ion selective electrode (ISE) (Radiometer ABL 90 FLEX Plus), reference range 1.14-1.34 mmol/L.

1. PTHrP Staining:

10 micron sections were cut from the surgical pathology specimens and stained following established protocols. Immunohistochemistry for PTHrP was performed using standard techniques. Briefly, paraffin embedded sections were deparaffinized using Histoclear (National Diagnostics) and rehydrated in graded alcohols. Antigen retrieval was performed in 7mM citrate buffer, pH 6.0 in a pressure cooker for 15 minutes. Sections were blocked with 10% normal goat serum in PBS with 0.2% triton, 0.05% tween 20 and 0.1% bovine serum albumin for 1 hour at room temperature, followed by incubation with rabbit-anti-PTHrP 1-14 antibody (gift of Dr. Jack Martin, Melbourne, Australia) for 1 hour at room temperature. Anti-PTHrP staining was detected using Vector ABC ELITE kit (catalogue # PK6101) and DAB (3,3'-diaminobenzidine, Vector Impact DAB SK 4105) following the manufacturer’s directions. Sections were lightly counterstained with diluted Harris Hematoxylin (Sigma HHS-16 ), dehydrated in graded alcohols followed by Histoclear and then cover-slipped and sealed with Cytoseal 60 (Fisher Scientific Co).
